# Functionality of Cellulose Nanofiber as Bio-Based Nucleating Agent and Nano-Reinforcement Material to Enhance Crystallization and Mechanical Properties of Polylactic Acid Nanocomposite

**DOI:** 10.3390/polym13030389

**Published:** 2021-01-27

**Authors:** Siti Shazra Shazleen, Tengku Arisyah Tengku Yasim-Anuar, Nor Azowa Ibrahim, Mohd Ali Hassan, Hidayah Ariffin

**Affiliations:** 1 Laboratory of Biopolymer and Derivatives, Institute of Tropical Forestry and Forest Products (INTROP), Universiti Putra Malaysia, Serdang 43400, Malaysia; shazra.shazleen@yahoo.com; 2 Department of Bioprocess Technology, Faculty of Biotechnology and Biomolecular Sciences, Universiti Putra Malaysia, Serdang 43400, Malaysia; ttengkuarisyah@gmail.com (T.A.T.Y.-A.); alihas@upm.edu.my (M.A.H.); 3 Department of Chemistry, Faculty of Science, Universiti Putra Malaysia, Serdang 43400, Malaysia; norazowa@upm.edu.my

**Keywords:** cellulose nanofiber, nucleating agent, crystallization rate, polylactic acid, organic nanofiller

## Abstract

Polylactic acid (PLA), a potential alternative material for single use plastics, generally portrays a slow crystallization rate during melt-processing. The use of a nanomaterial such as cellulose nanofibers (CNF) may affect the crystallization rate by acting as a nucleating agent. CNF at a certain wt.% has been evidenced as a good reinforcement material for PLA; nevertheless, there is a lack of information on the correlation between the amount of CNF in PLA that promotes its functionality as reinforcement material, and its effect on PLA nucleation for improving the crystallization rate. This work investigated the nucleation effect of PLA incorporated with CNF at different fiber loading (1–6 wt.%) through an isothermal and non-isothermal crystallization kinetics study using differential scanning calorimetry (DSC) analysis. Mechanical properties of the PLA/CNF nanocomposites were also investigated. PLA/CNF3 exhibited the highest crystallization onset temperature and enthalpy among all the PLA/CNF nanocomposites. PLA/CNF3 also had the highest crystallinity of 44.2% with an almost 95% increment compared to neat PLA. The highest crystallization rate of 0.716 min^–1^ was achieved when PLA/CNF3 was isothermally melt crystallized at 100 °C. The crystallization rate was 65-fold higher as compared to the neat PLA (0.011 min^–1^). At CNF content higher than 3 wt.%, the crystallization rate decreased, suggesting the occurrence of agglomeration at higher CNF loading as evidenced by the FESEM micrographs. In contrast to the tensile properties, the highest tensile strength and Young’s modulus were recorded by PLA/CNF4 at 76.1 MPa and 3.3 GPa, respectively. These values were, however, not much different compared to PLA/CNF3 (74.1 MPa and 3.3 GPa), suggesting that CNF at 3 wt.% can be used to improve both the crystallization rate and the mechanical properties. Results obtained from this study revealed the dual function of CNF in PLA nanocomposite, namely as nucleating agent and reinforcement material. Being an organic and biodegradable material, CNF has an increased advantage for use in PLA as compared to non-biodegradable material and is foreseen to enhance the potential use of PLA in single use plastics applications.

## 1. Introduction

Today, most plastics are designed to be discarded after single use, leading to the accumulation of single-use disposable plastics waste that ends up in landfills, dumps, or in the open environment. Commonly, single-use plastics are used for packaging and carry bags. Plastics are mainly composed of polymers and other chemicals such as stabilizers, colorants, and processing aids in which the quantity and type depend on the processing method and targeted applications [1]. Consumer plastics are generally made of petroleum-based polymers, and most of the plastics currently used are non-biodegradable [2]. This has contributed to the environmental issue due to mismanagement of plastics [3].

Biodegradable plastics can be used as an alternative to the currently non-biodegradable single-use plastics. Biodegradable plastics can be degraded in nature with the aid of microorganisms. Complete degradation of the plastics produces carbon dioxide and water without introducing toxic materials to the environment [4]. Considerable effort has been made to develop bioplastic/biopolymer from biodegradable resources. Polylactic acid (PLA) is an example of bioplastic which has attracted interest from industries due to its potential related to its superior properties.

PLA has significantly lower crystallization rate as compared to conventional plastics such as polyethylene (PE) and polypropylene [5]. This may limit its melt processability (e.g., extrusion and injection molding), as slow crystallization may imply longer molding cycle time, and contributes to the low productivity and high energy consumption during production [6,7]. In regard to this matter, the addition of nucleating agent into the PLA is seen as one of the effective approaches for the purpose of addressing slow solidification time after thermal processing. Nucleation of polymer depends on the properties of the nucleating agent used, such as particle size, geometry, surface structure, and interfacial interactions with the polymer matrix [8]. Several studies have been conducted to identify the effective nucleating agent for PLA, such as graphene [7,9], talc [10,11,12], and natural fiber [13,14,15,16]. Generally, smaller particles make a more effective nucleating agent than larger particles due to their large specific surface areas [17,18].

To broaden the utilization of PLA for biodegradable-based product development, the addition of biodegradable nucleating agents with excellent nucleation ability is needed. Recently, studies have focused on the use of cellulose nanofiber (CNF) to promote crystallization in PLA by acting as a heterogeneous crystal nucleating agent [19]. CNF can accelerate the process of crystallization by increasing the number of crystal nuclei and reducing the crystallization half-time [10]. In fact, CNF has been widely utilized owing to its outstanding properties, namely high flexibility, good mechanical strength, high crystallinity, and high aspect ratio, which are advantageous to enhance the mechanical properties of polymers [20,21]. The nanometric scale effect and large specific surface area of nanomaterials have particularly helped to improve the properties of PLA and make the PLA nanocomposites more prominent compared to neat PLA [22]. Ariffin et al. [23] revealed that the addition of 3 wt.% of CNF to PLA increased both the tensile strength and Young’s modulus by 13 and 37%, respectively, compared to the neat PLA. The improvement in mechanical properties of the PLA/CNF could be related to the high crystallinity of CNF, as they also revealed that the addition of 3 wt.% CNF was able to increase the crystallinity index of nanocomposite by 14% compared to the neat PLA. Similar findings were also reported by Jonoobi et al. [24], Nguyen et al. [25], and Norrrahim et al. [26].

Despite studies on the use of CNF as a nucleating agent or reinforcement material, there is lack of information on the correlation between the amount of CNF in PLA that promotes its functionality as reinforcement material, and its effect on PLA nucleation for improving the crystallization rate. Hence, this study attempted to investigate the dual role of CNF as nucleating agent and reinforcement material in PLA by analyzing the correlation effect of the CNF weight ratio on both the crystallization and mechanical performances. Crystallization kinetics behavior was determined by differential scanning calorimetry (DSC) analysis through isothermal and non-isothermal methods.

## 2. Materials and Methods

### 2.1. Materials

Polylactic acid (PLA) (grade 2003D, NatureWorks LLC, Minnetonka, MN, USA) in pellet form was purchased from Ecoscience Sdn. Bhd (Selangor, Malaysia) with a melt flow index of 6.0 g/10 min at 210 °C was used as is. The number and weight-average molecular weights (M_n_ and M_w_, respectively) and polydispersity index (PDI; calculated as the ratio of M_w_/M_n_) of 165,189 g/mol, 76,066 g/mol, and 2.17, respectively, were determined by gel permeation chromatography (GPC) at 40 °C using THF as eluant on a Waters apparatus (Waters, MA, USA) equipped with three columns, Styragel HR1, HR3, and HR5, and with a Waters 2414 refractive index detector at an elution rate of 1 mL/min. The system was calibrated using polystyrene standards. Cellulose nanofiber (CNF) with a concentration of 2 wt.% fiber slurry was purchased from ZoepNano Sdn. Bhd (Selangor, Malaysia).

### 2.2. Methods

#### Preparation of PLA/CNF Nanocomposites

Neat PLA and PLA/CNF nanocomposites were melt-blended using a Brabender Plasticoder (Brabender Messtechnik GmbH Co., Duisburg, Germany) internal mixer. Prior to mixing, PLA pellets were dried under vacuum at 60 °C for 24 h to remove moisture, because it is very essential to minimize the hydrolytic degradation during processing at high temperatures. The dried PLA pellets were mixed with CNF at different contents (1–6 wt.%) at 170 °C for 30 min with a rotor speed of 70 rpm. Neat PLA and PLA/CNF nanocomposites were then compression molded using a hydraulic hot press at 160 °C, 110 kg cm^−2^, and 10 min molding temperature, pressure, and time, respectively. Cooling was then performed for 5 min under same pressure.

### 2.3. Characterization

#### 2.3.1. Crystallization Kinetics Analysis

Non-isothermal and isothermal crystallization behaviors of the nanocomposites were characterized using DSC (Q200, TA Instruments, New Castle, DE, USA). Indium was used as standard calibration for temperature and heat of fusion. Sample weighing between 7 and 10 mg was used for the measurement.

For non-isothermal crystallization, the samples were first heated from 30 °C to 190 °C at a rate of 2 °C/min and maintained at this temperature for 3 min to remove the prior thermal history of the samples. The samples were then cooled to −40 °C at the same rate and held at that temperature for 3 min to evaluate their ability to crystallize upon cooling. Subsequently, the samples were reheated to 190 °C at the same rate.

For isothermal crystallization, the samples were firstly heated to 190 °C at a rate of 10 °C/min and maintained at this temperature for 3 min to remove their prior thermal history. The samples were then rapidly cooled to the isothermal crystallization temperature of 90, 100, and 110 °C at a rate of 50 °C/min and held at this temperature until the crystallization process in the samples was completed.

#### 2.3.2. Mechanical Analysis

Tensile strength (MPa), elongation at break (%), and Young’s modulus (GPa) were measured by using an Instron 5566 Universal Testing Machine (Norwood, MA, USA) with a load cell of 10 kN and a crosshead speed of 5 mm/min at room temperature. Five dog-bone shaped specimens, each with a 3 mm thickness, were tested according to the standard method of ASTM D 638-05.

#### 2.3.3. X-ray Diffraction Analysis

The size of spherulite was measured using X-ray diffraction (XRD) analysis. The experiment was performed using an automated Shimadzu 6000 X-ray diffractometer (Tokyo, Japan) operating at 40 kV, current of 20 mA, and Cu radiation of λ = 1.5406 Å between 2θ = 5–50° at a scan rate of 2 °/min. A method based on the Scherrer equation was used to estimate the spherulite size from the broadening of the diffraction pattern. The crystallite dimension was calculated by the following equation:

(1) $$D=\frac{K\lambda }{\beta \cos\theta }$$

where D is the crystallite size; K is the Scherrer constant, which is 0.94; λ is the wavelength of the X-rays in nm; β is the full width half maximum; and θ is the Bragg angle in radians.

#### 2.3.4. Morphological Analysis

The morphology of the fracture surfaces of the PLA/CNF nanocomposites from tensile testing were observed using a field emission scanning electron microscope (FESEM) (FEI Nova NanoSEM 230, Hillsborough, OR, USA). The acceleration voltage used was 5 kV, and samples were sputter-coated with gold prior to FESEM observation to avoid charging.

## 3. Results and Discussion

### 3.1. Non-Isothermal Crystallization Kinetics

**Figure 1:**
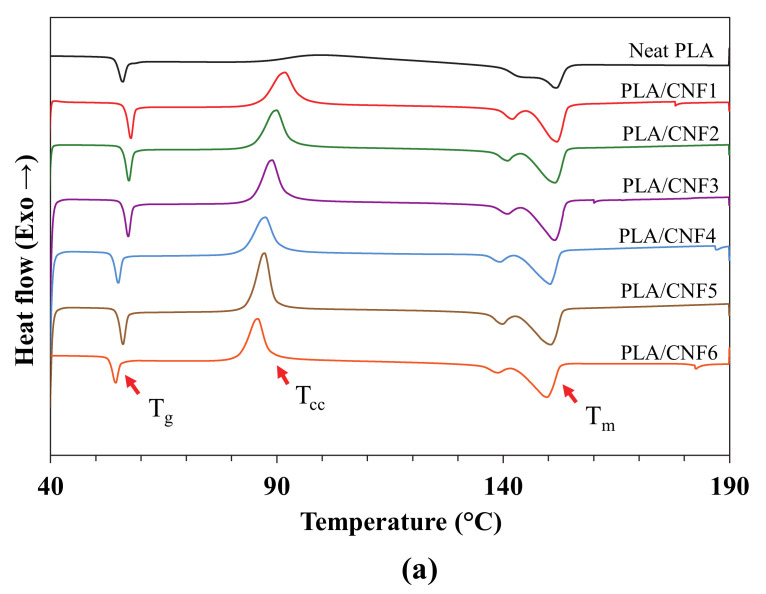

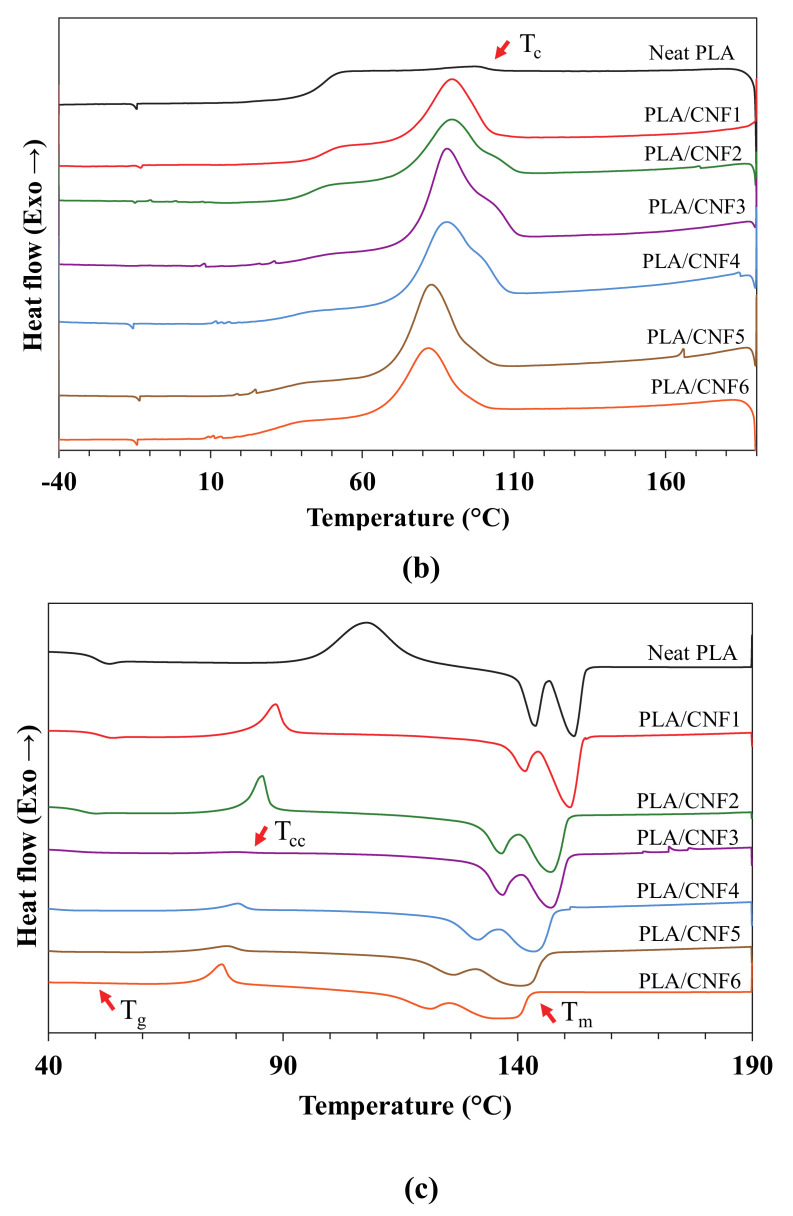
DSC curves at 2 °C/min for neat PLA and PLA/CNF nanocomposites: (**a**) first heating scan, (**b**) cooling scan, and (**c**) subsequent heating scan.

The nucleation effect of different CNF content on the crystallization kinetics of PLA/CNF nanocomposites was determined using DSC measurements. Figure 1b,c shows non-isothermal DSC cooling curves and subsequent heating curves of neat PLA and PLA/CNF (1–6 wt.%). Table 1 summarizes the thermal properties of nanocomposites estimated from the DSC curves, including glass transition temperature (T_g_), crystallization peak temperature (T*_c_*), cold crystallization peak temperature (T*_cc_*), melting peak temperatures (T_m1_, T_m2_), enthalpy of crystallization (∆H_c_), enthalpy of cold crystallization (∆H*_cc_*), enthalpy of fusion (∆H*_m_*), and degree of crystallinity (X_c_). The degree of crystallinity (X_c_) for neat PLA and PLA/CNF nanocomposites were calculated as follows:

(2) $$X_{c}\;=\;\frac{∆Hm\;-\;∆Hcc}{\;∆H_{m}^{0}}\;\times \;100\%$$

where ∆H*_m_* is the enthalpy of melting, ∆H*_cc_* is the crystallization enthalpy during the DSC scan, and ∆H°*_m_* is the enthalpy of melting of 100% crystalline PLA (∆H°*_m_* of PLA = 93.7 J g^−1^).

Data tabulated in Table 1 and DSC curves in Figure 1b show that the crystallization peak of neat PLA was almost unseen and exhibited low crystallization enthalpy, ∆H_c_. Furthermore, all PLA-reinforced CNF nanocomposites had a clear and sharper crystallization peak upon cooling. It was observed that the onset crystallization temperature, T_c_, and ∆H_c_ increased as CNF loading increased from 1 to 3 wt.%. The T_c_ increment was about 9 °C from 106 °C (neat PLA) to 115 °C after the reinforcement with 3 wt.% CNF. The higher tendency of PLA for earlier crystallization at higher CNF loading up to 3 wt.% may be due to good dispersion and well-distributed CNF at those ratios in the PLA matrix that could produce a much stronger nucleation effect to initiate the crystallization process to occur [27]. However, further increments in CNF loading (4–6 wt.%) reduced the T_c_ and ∆H_c_. This might be due to the agglomeration of CNF at higher loading that hinders the mobility of PLA chains and consequently, inhibits the crystallization of the polymer chains in forming spherulites [28].

In the subsequent heating process, as shown in Figure 1c and Table 1, the glass transition temperature, T_g_, for neat PLA was observed at 50.4 °C. It was seen that the addition of CNF into PLA matrix reduced the T_g_. The increase in CNF loading may have exerted some influence on the increased PLA chain mobility in nanocomposite samples, which resulted in better flexibility [29]. However, it should be noted that one of the key factors in the variability of T_g_ is the polymeric chain mobility and not necessarily correlated with the change in material crystallinity [30].

Furthermore, a second exothermic peak observed in Figure 1c corresponds to the peak of cold crystallization temperature, T_cc_. A very clear cold crystallization peak was observed for neat PLA in the subsequent heating phase, which demonstrated that this material had undergone a slow crystallization process as it was unable to crystallize properly during cooling. On the other hand, the cold crystallization peak of the samples shifted to lower temperatures and exhibited lower cold crystallization enthalpy with the presence of CNF in the PLA matrix. Reinforcing PLA with low composition of CNF (1–3 wt.%) resulted in smaller cold crystallization peaks, in which the peak for 3 wt.% CNF-reinforced nanocomposite was almost unseen. This indicated that the complete crystallization process occurred during the cooling process, mainly due to the CNF nucleation effect. In contrast, a clear cold crystallization peak for PLA reinforced with 4–6 wt.% CNF was seen to indicate some incomplete crystallization process during cooling. This finding is in agreement with the previous studies related to the CNF nucleation effect on the thermal properties of PLA and PLA reinforced with natural fiber [31,32,33,34,35].

For melting behavior, neat PLA and PLA nanocomposites showed bimodal melting peaks, as shown in Figure 1c, with T_m1_ at the first melting point (at a lower melting temperature) and T_m2_ at the subsequent peak (at a higher melting temperature). The first peak was observed at around 121–143 °C and the other at 135–152 °C. The double endothermic peaks were attributed to melting-recrystallization–melting processes of PLA lamellae. The first endothermic peak was related to the melting of thin lamellae developed during heating process, while the second peak was related to the melting of lamellae formed through the melting–recrystallization of primary thin lamellae at higher temperatures [36]. Moreover, PLA can crystallize into three polymorphic forms which are α, β, and γ depending on the process conditions; the α structure is more stable than β and can be formed from the molten state [37]. This literature also reports the presence of an α crystalline phase with a looser and disorderly structure known as α’, which fuses at lower melting temperature (T_m1_) and then recrystallizes in a more stable form α, which is subsequently fused at higher melting temperature (T_m2_). Hence, this explains the presence of double exothermic melting peaks in the thermograph above.

Previous studies conducted by Kanig et al. [38] and Ding et al. [39] reported that the crystallites size distribution in the polymer can also be estimated from the DSC data. It can be measured from the melting phase of nanocomposites, where it is directly proportional to the width of the melting peak. The relatively broad melting range corresponds to the wide distribution of crystallite sizes in the polymer matrix, while the narrow peak indicates narrow crystallite size distribution. In this study, the widest crystallite size distribution was observed in PLA/CNF6 nanocomposite with a melting peak span of ∆T_m_ = 42.4 °C, whereas neat PLA had the narrowest distribution size of crystallites with ∆T_m_ = 28.5 °C. The increase in CNF loading could have contributed to the increase in crystal density and thus, produce wider crystallites size distribution. This could be attributed to the CNF presence in the PLA matrix, which greatly facilitated the nucleation of crystallites for PLA and could serve as a heterogeneous nucleation site for crystallization.

The degree of crystallinity, X_c_, of the nanocomposites could also be prompted by the incorporation of CNF. The X_c_ of neat PLA was only 2.3% at a low scan rate of 2 °C/min, whereas the addition of CNF in the matrix enhanced the value of X_c_. The PLA/CNF3 nanocomposite in particular had the highest crystallinity of 44.2% with an almost 95% increment relative to neat PLA. At a low scan rate, the PLA would have sufficient time to crystallize in the cooling phase, thus inhibiting the formation of cold crystallization peak at the subsequent heating step. This suggests that the melt crystallization cycle was completed during the prior cooling phase. This was highly attributable to the property of PLA as a semi-crystalline polymer with low crystallization ability and slow crystallization rate, in which a higher cooling rate causes the crystal growth to significantly decrease, since PLA molecules do not have enough mobility to diffuse to the crystallite. In fact, they freeze in their high entropic state leading to minimal crystallinity [40]. Therefore, the 2 °C/min scan rate was performed, and the thermograph is presented in Figure 1. This explanation was in agreement with the findings reported by Lu et al. [41], where a rate of 2 °C/min for all specimens showed higher crystallinity compared to 5, 10, and 20 °C/min.

### 3.2. Isothermal Crystallization Kinetics

Isothermal crystallization behavior of the neat PLA and PLA/CNF nanocomposites was also investigated to fully understand the nucleation effect of different CNF loading on the crystallization rate of PLA. Li and Huneault [42] reported that PLA crystallized at a temperature range of 80 °C to 120 °C. Based on the non-isothermal cooling curves in Figure 1b, it was shown that the nanocomposites have an average onset T_c_ at 100 °C. Hence, the isothermal crystallization study was conducted at 90, 100, and 110 °C.

**Figure 2:**
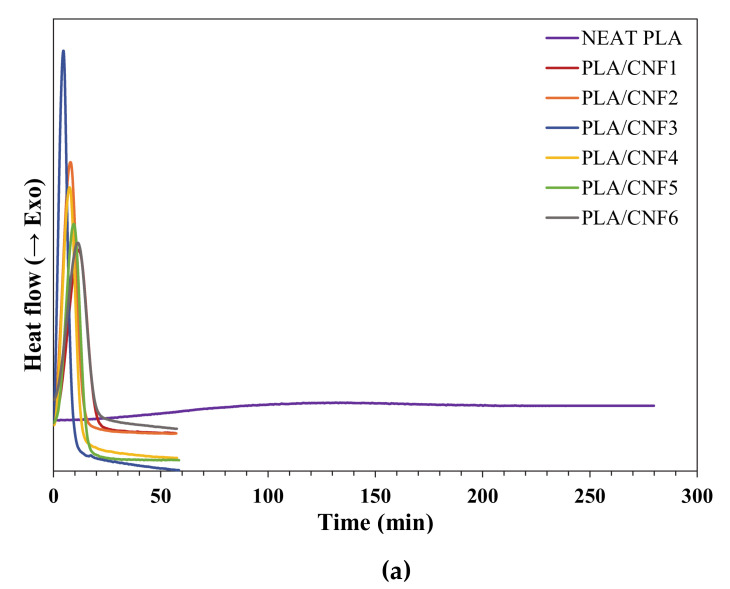

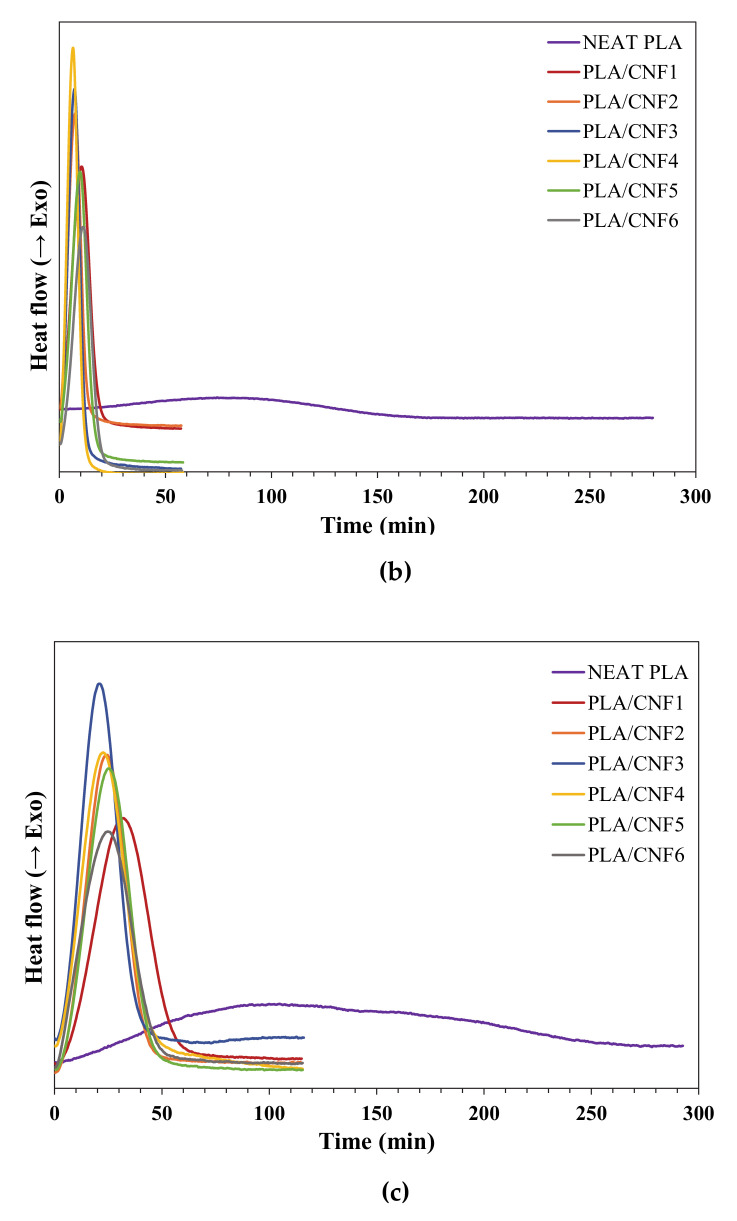
Isothermal crystallization isotherms of neat PLA and PLA/CNF nanocomposites at (**a**) 90, (**b**) 100 and (**c**) 110 °C.

DSC curves of isothermal crystallization for neat PLA and PLA/CNF nanocomposite samples are presented in Figure 2. At T_c_= 90, 100, and 110 °C, it is obvious that the crystallization rate of neat PLA was extremely slow, in which crystallization peak was hardly observed even after 100 min. The addition of CNF generally resulted in the appearance of the sharp exothermal peak and shifted the crystallization towards a shorter timeframe, which was within 30 min. The finding indicates that CNF induced faster crystallization in PLA, thus accelerating the crystallization speed.

To analyze the isothermal crystallization kinetics, the isothermal DSC curves were integrated between *t* = 0 and *t* and divided by the overall crystallization rate to calculate relative degree of crystallinity as follows:

(3) $$X_{\mathrm{rel}}\;=\;\frac{\int_{0}^{t}\frac{dH\left(t\right)}{dt}\;dt}{\int_{0}^{\infty }\frac{dH\left(t\right)}{dt}\;dt}$$

**Figure 3:**
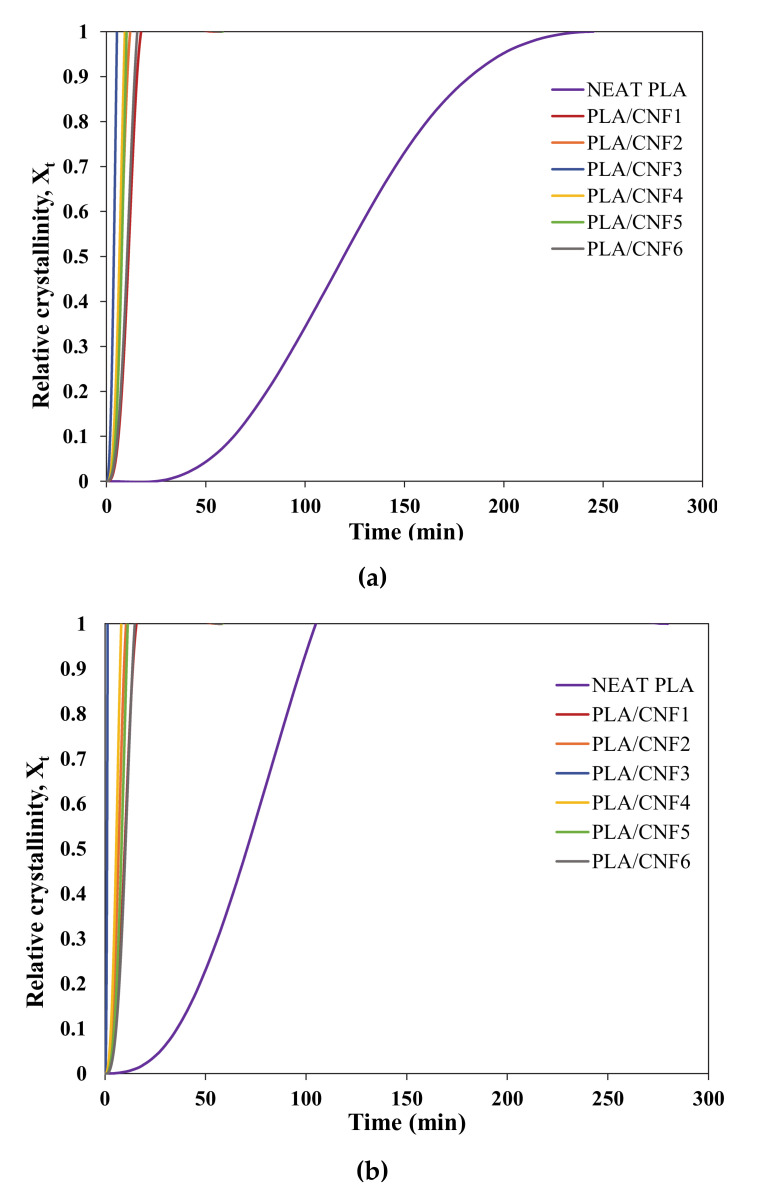

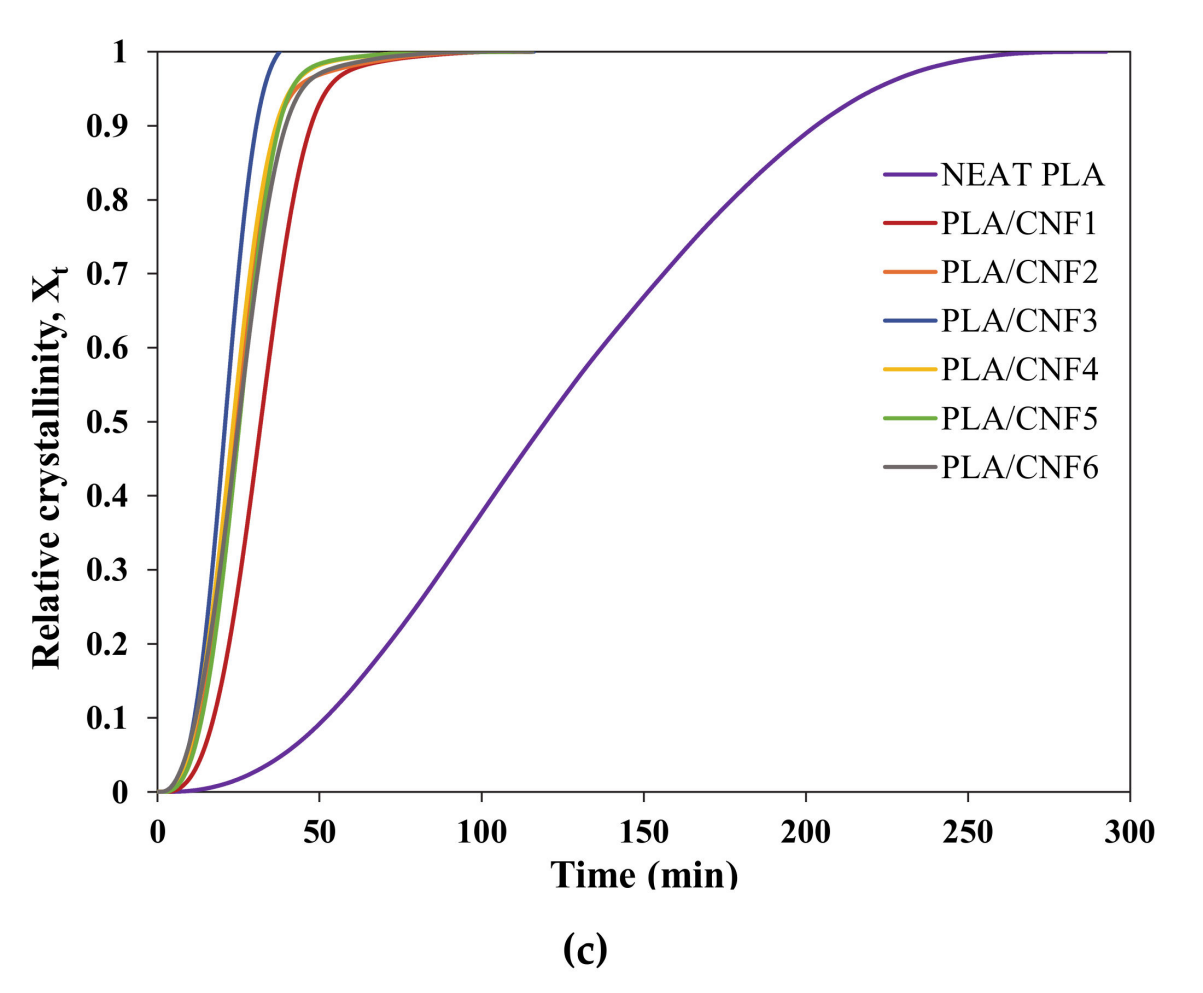
The relative crystallinity of neat PLA and PLA/CNF nanocomposites at (**a**) 90, (**b**) 100, and (**c**) 110 °C.

**Figure 4:**
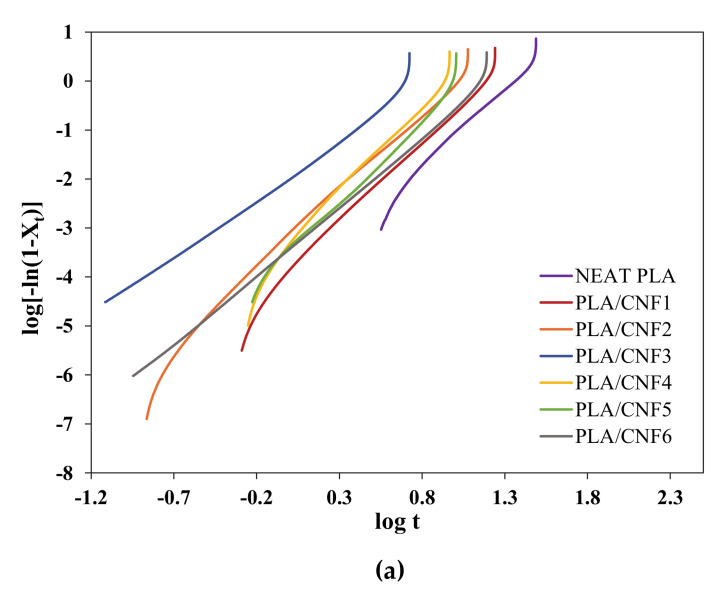

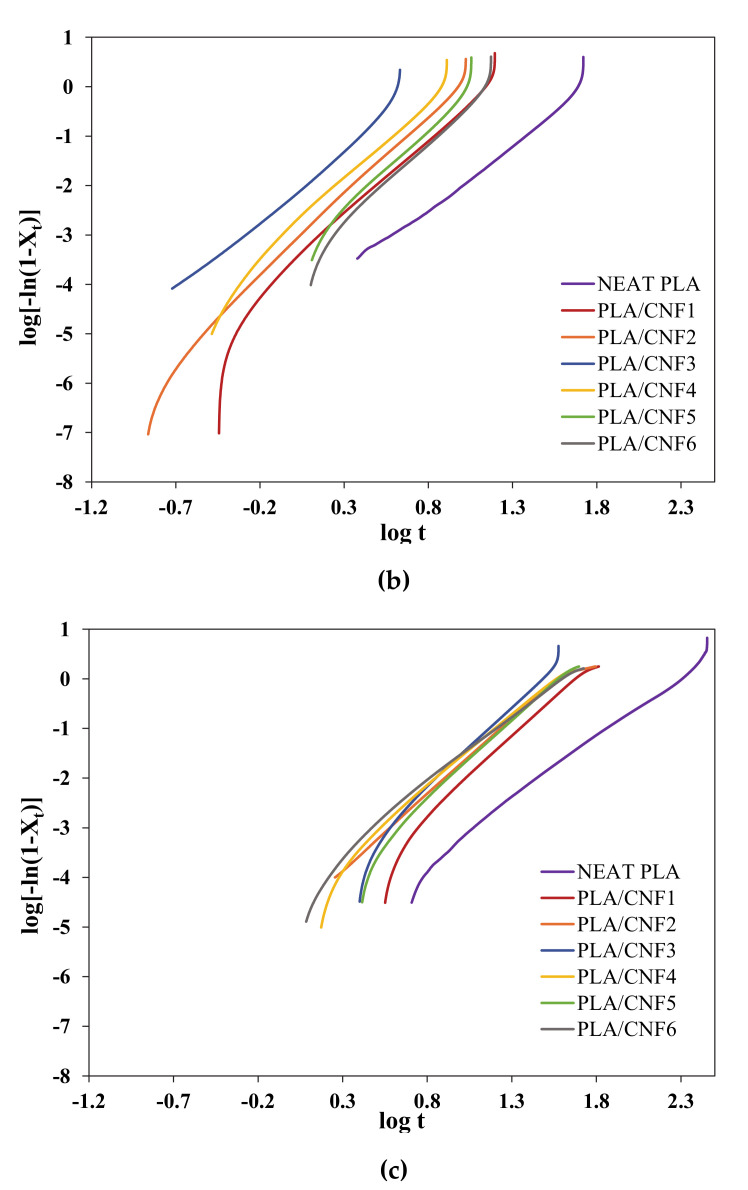
Avrami plots of neat PLA and PLA/CNF nanocomposites isothermally crystallized at (**a**) 90, (**b**) 100, and (**c**) 110 °C.

The Avrami equation was used to study the isothermal melt crystallization kinetics, where relative degree of crystallinity (X_rel_) is described as follows:

X_rel_ (t) = 1 − exp (−*kt^n^*) (4)

where *n* is the Avrami exponent that depends on the nature of the nucleation mechanism and growth geometry of the crystal, *k* is the crystallization rate constant that involves both nucleation and growth rate parameters, and *t* is time. Equation (3) can be transformed into the double-logarithmic form,

log (−ln (1 − X_rel_(t))) = log *k* + *n* log t (5)

where the parameters *n* (slope) and *k* (y-intercept) are determined by plotting log (−ln (1 − X_rel_(t))) against log *t*. Figure 3 and Figure 4 shows the X_t_ versus *t* and Avrami plots for neat PLA and PLA/CNF (1–6 wt.%) nanocomposites isothermally melt-crystallized at 90, 100, and 110 °C. The crystallization half time, t_1/2_, is another important crystallization kinetics parameter, which is defined as the time required to achieve 50% of the final crystallinity of the samples and was calculated by the following equation:

(6) t1/2= ln 2k1n

Generally, the crystallization rate is expressed through the use of crystallization half time and can be calculated by the reciprocal of *t_1/2_*.

(7) $$\mathrm{Crystallization}\;\mathrm{rate}\;=\;\frac{1}{t_{1/2}}$$

Avrami parameters calculated from the plots in Figure 4 are summarized in Table 2. The *n* value usually takes an integer number between 1 and 4, but due to secondary crystallization, the *n* value adopted fractional numbers [43]. As seen from Table 2, the *n* values were around 2.4–3.6, suggesting three-dimensional crystallization growth and two growth mechanisms were present: two-dimensional at the onset of the crystallization process with the formation of two-dimensional lamellar-shaped crystals, and three-dimensional growth during further growth of spherulite formation [44,45]. Similar *n* values were reported for PLA/nucleating agent (MFC, CNC, talc, and uracil) composites by other studies, where the Avrami exponent *n* is in the range of 2.8–4.9 [44,45,46,47,48]. Moreover, the *k* value for neat PLA and PLA/CNF nanocomposites increased with the T_c_ and then decreased after reaching a maximum value at 100 °C due to the difficulty of crystal nucleation at elevated temperature [49].

**Figure 5:**
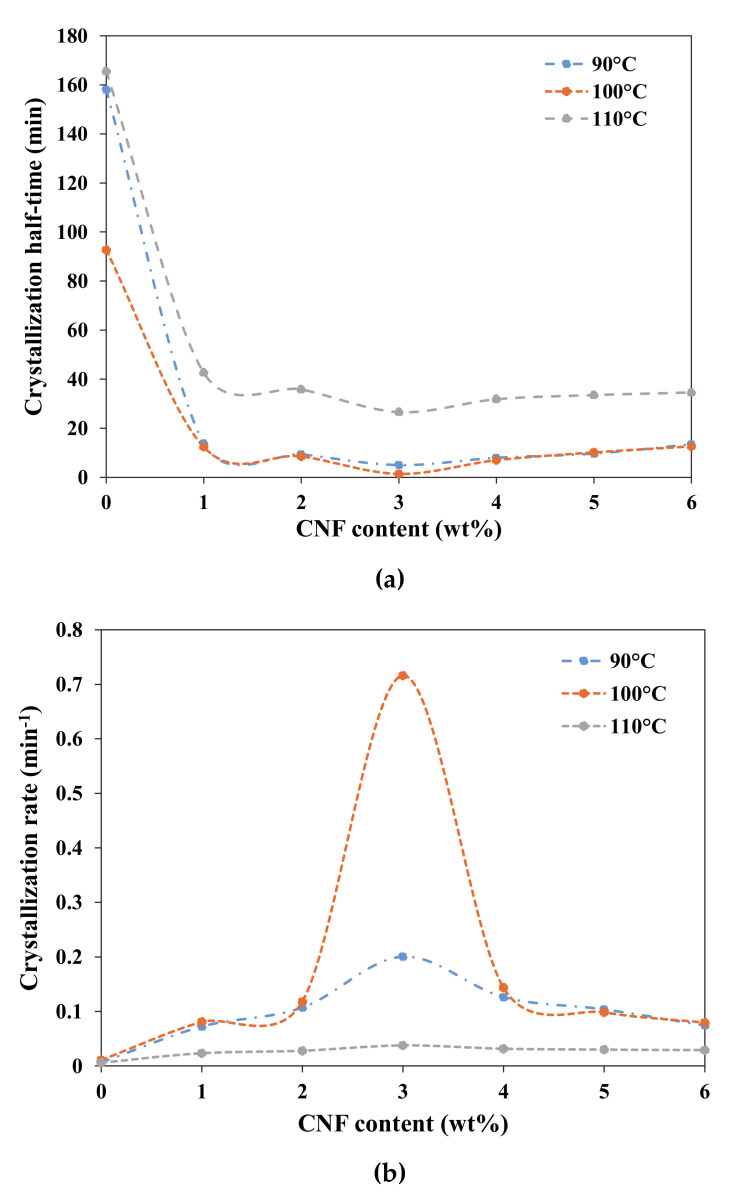
Dependence of (**a**) crystallization half-time and (**b**) crystallization rate on CNF content for PLA melt-crystallized isothermally at 90, 100, and 110 °C.

Figure 5a,b represents the correlation between CNF content with crystallization half-time and crystallization rate for PLA melt-crystallized isothermally at 90, 100 and 110 °C. As seen in Table 2 and Figure 5a, incorporation of CNF up to 3 wt.% effectively reduced the t_1/2_ values to 4.99, 1.40 and 26.63 min from the 158.01, 92.72 and 165.59 min of the neat PLA, respectively, when isothermally crystallized at 90, 100 and 110 °C. This proves the nucleating property of CNF that leads to faster crystallization. Nonetheless, CNF content beyond 3 wt.% resulted in an increment of t_1/2_ values for all T_c_ that may be attributed to CNF agglomeration at high loading, thus limiting the ability of CNF to act as nucleating agent. Tri et al. [50] also reported that the same phenomenon occurred by increasing talc loading for PLA/PHB composites. It can therefore be assumed that CNF loading has a significant effect on the crystallization rate of PLA.

Moreover, among the T_c_ investigated, more distinct nucleation effects with a minimum t_1/2_ value (i.e., a maximum in crystallization rate) could be seen at T_c_ = 100 °C for all the samples. This is because this temperature corresponds to the optimum temperature of isothermal crystallization of the samples. The t_1/2_ value increased on both sides towards the melting points at high temperature and the glass transition at low temperature for neat PLA and PLA-reinforced CNF nanocomposites. This finding is consistent with data obtained in the previous research conducted by Pan et al. [47], whereby the neat PLLA and PLA/1% uracil showed more distinct nucleation effects at T_c_ = 100–110 °C than those at low T_c_ (e.g., 80–90 °C) and high T_c_ (e.g., 120–140 °C). The authors explained that it is a typical characteristic of polymer crystallization, because the process is based on nucleation-controlled crystallization and diffusion-controlled crystallization at low and high supercooling.

Besides that, the crystallization rate of PLA-reinforced CNF nanocomposites was higher than that of the neat PLA, as illustrated in Figure 5b. This finding confirms that the presence of CNF accelerates the isothermal melt crystallization of PLA. The highest crystallization rate of 0.716 min^−1^ was achieved when 3 wt.% of CNF was reinforced into the PLA matrix and isothermally melt crystallized at T_c_ = 100 °C. This was 65 fold higher than the neat PLA.

### 3.3. Mechanical Performance

Mechanical testing was performed to evaluate the effect of CNF addition on the mechanical properties of PLA/CNF nanocomposites. The results for neat PLA and PLA nanocomposites are shown in Table 3.

Mechanical analysis revealed that the incorporation of 1 to 4 wt.% CNF to PLA significantly increased both tensile strength and Young’s modulus of nanocomposites. The tensile strength of PLA increased by 7% from 70.6 MPa (neat PLA) to 76.1 MPa with the addition of 4 wt.% CNF. This result indicates good transfer of load from PLA matrix to the CNF [51,52]. However, a reduction in these two properties was observed when CNF loading was more than 4 wt.%. The tensile strength of PLA/CNF5 and PLA/CNF6 nanocomposites decreased by 9 and 11%, respectively. This was mainly due to the agglomeration of CNF at higher loading having a reversal effect on the mechanical properties. This was supported by Eyholzer et al. [53] which discussed that agglomerates can act as stress concentrators and can impede mechanical properties as they reduce the effective surface area of the particles and increase the interparticle distance, thereby counteracting the nanosize effect. This observation was also expected, since the crystallinity of a material is directly proportional to tensile strength.

Young’s modulus also exhibited a similar trend as tensile strength, where PLA/CNF nanocomposites showed an increment in Young’s modulus with increasing CNF content. Improvement in nanocomposite stiffness can be associated with the restrictions on PLA mobility chains introduced by the presence of CNF at higher loading [25]. Most studies agreed that the addition of CNF can enhance the mechanical properties of PLA composites tremendously. Ariffin et al. [23] revealed that both tensile strength and Young’s modulus increased up to 13 and 27%, respectively, after 3 wt.% CNF-OPMF was reinforced into PLA. This finding is in agreement with the previous studies conducted by Gitari et al. [29], in which the hydrated softwood purified CNF was reinforced into PLA through the solvent-casting method. The findings revealed that reinforcing by 1 wt.% CNF could increase both the tensile strength and Young’s modulus by 17%. Similarly, Safdari et al. [54] also reported that the tensile strength and Young’s modulus of PLA/5 wt.% CNF were higher by 31 and 50%, respectively, compared to the neat PLA. Besides that, CNF had a large specific surface area due to its small size and was found to be a heterogeneous nucleation site for crystallization in PLA. The improvement in mechanical properties of the PLA/CNF nanocomposites could also be related to the high crystallinity of CNF, as the addition of 3 wt.% CNF was able to increase the crystallinity of nanocomposites with an almost 95% increment relative to neat PLA, as discussed previously in Section 3.1. It should be emphasized that increasing the crystalline phase of a polymer may eventually increase the mechanical properties of the composite, since it creates crosslinking sites that improve the stiffness of the composite [18]. These findings prove the dual role of CNF as a nucleating agent that simultaneously acts as nano-reinforcement material in enhancing the crystallization and mechanical properties of PLA.

On the other hand, elongation at break was slightly reduced by increasing the CNF loading. This was attributed to the CNF properties itself. The nanocomposites became more brittle because the CNF could only provide a strengthening effect but not an elongating effect [51]. Indeed, CNF could enhance the crystallinity of PLA, but poor dispersion of CNF and lack of interfacial adhesion between CNF and PLA particularly at higher loading limited the mechanical strength of PLA/CNF nanocomposites.

### 3.4. Spherulite Size

**Figure 6:**
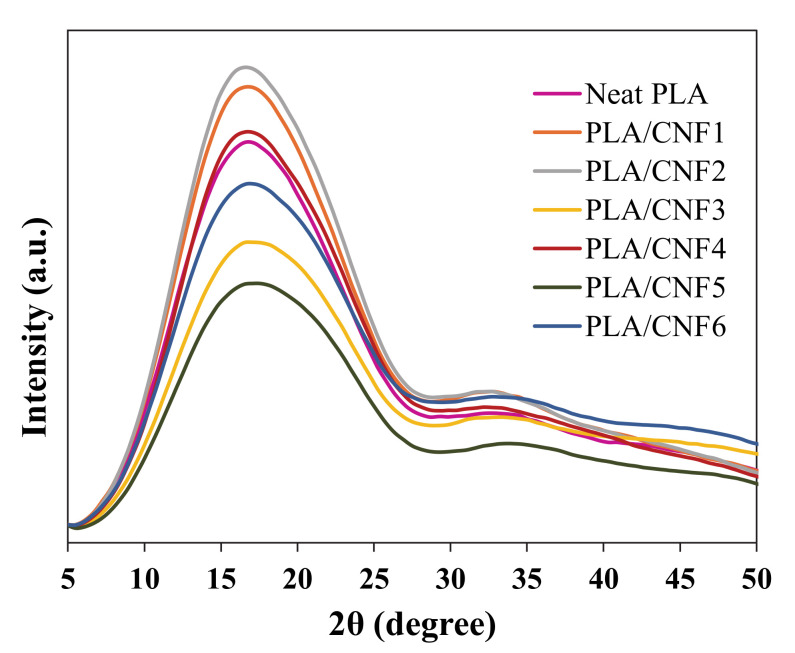
XRD diffraction patterns of neat PLA and PLA/CNF nanocomposites.

Figure 6 displays the XRD patterns of neat PLA and PLA/CNF nanocomposites with various CNF contents, and the crystallite sizes obtained are tabulated in Table 4. β represents full width half maximum (FWHM) of peak, while D is the crystallite size.

This analysis revealed that one of the tested samples exhibited some peaks corresponding to a crystalline structure. All nanocomposite samples exhibited low intensity and showed a peak position towards lower angles. This observation may be due to a low degree of order and deformation in the crystalline structure. Therefore, it is obvious that all nanocomposite crystallites adopted the distorted α’-form orthorhombic crystal formation [40]. In neat PLA, the crystallite size was found to be 0.761 nm. It was exhibited that reinforcing CNF into the PLA matrix did not change the crystallites size much differently as compared to neat PLA.

### 3.5. Morphological Analysis

**Figure 7:**
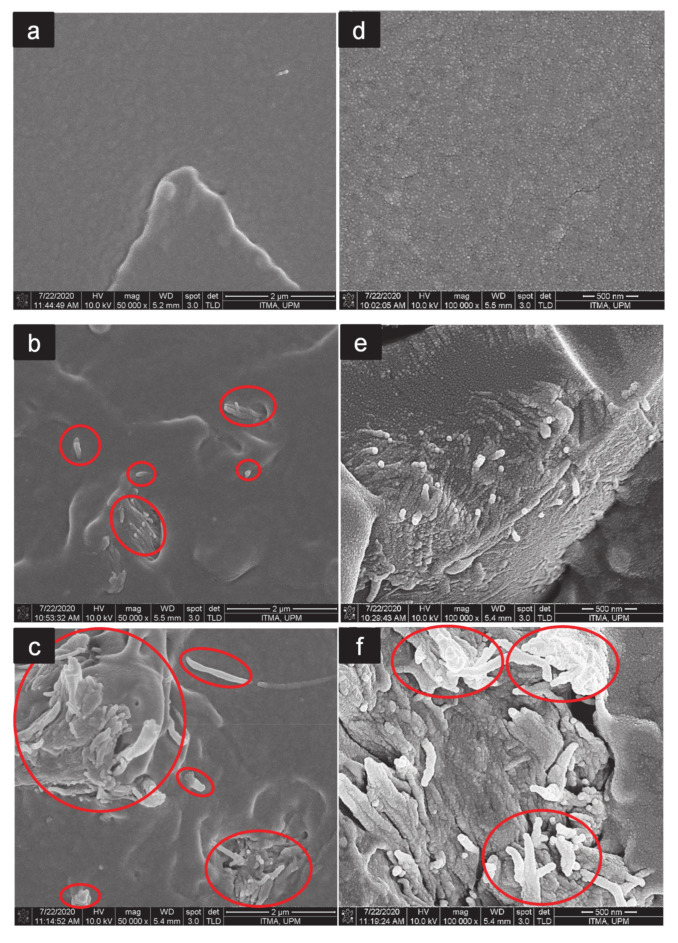
FESEM viewing at 50,000× and 100,000× magnification of the fractured surfaces of neat PLA (**a**,**d**), PLA/CNF3 (**b**,**e**), and PLA/CNF6 (**c**,**f**). Red circles show cellulose nanofibrils. PLA/CNF6 exhibits agglomerated nanofibrils.

The FESEM micrographs of the nanocomposites fractured surfaces, i.e., neat PLA (Figure 7a,d), PLA/CNF3 (Figure 7b,e), and PLA/CNF6 (Figure 7c,f) are shown, respectively.

This morphological analysis was conducted to observe if there was any visible CNF agglomerations in the fractured surfaces. As shown in Figure 7a,d, a relatively smooth surface could be seen in the neat PLA micrograph, showing typical brittle fracture characteristics [55]. For PLA/CNF3, some fiber breakages on the fractured surface could be observed and no agglomeration could be detected. This indicates that CNF was homogenously blended in the PLA matrix, which explains the high tensile properties of PLA/CNF nanocomposites. Nevertheless, a visible agglomeration was observed on PLA/CNF6 nanocomposites, as shown in Figure 7c,f (as indicated by the circles). Compatibility reduction between PLA and high contents of CNF might be one of the reasons behind the occurrence of agglomeration. As CNF loading increased, CNF agglomerated together and formed larger clusters instead of binding with PLA, hence resulting in poor interfacial adhesion between CNF and PLA, and thus explaining the lower tensile strength of PLA/CNF nanocomposites at higher CNF loading (beyond 3 wt.% of CNF) [35].

## 4. Conclusions

The effect of CNF as a nucleating agent for improving the crystallization and mechanical properties of PLA nanocomposites were investigated in this study. The function of CNF as a nucleating agent was evident as shown by the marked increment of crystallization rate to 0.716 min^−1^ when 3 wt.% CNF was used as compared to 0.011 min^−1^ for neat PLA during isothermal melt crystallization at T_c_ = 100 °C. This was 65-fold higher as compared to the neat PLA. In terms of mechanical properties, the reinforcement of 1–4 wt.% CNF increased the tensile properties of PLA. These findings affirm the role of CNF as an effective nucleating agent that simultaneously acts as a nano-reinforcement material in enhancing the crystallization and mechanical properties of PLA. Findings from this research suggest the potential of CNF as a nanofiller in biopolymer, which can be advantageous in the development of biodegradable single use plastics due to its biodegradable property. Research on the function of CNF in the kinetics of biopolymer biodegradation is currently in progress.

## Figures and Tables

**Table 1:** Non-isothermal crystallization data of neat PLA and PLA/CNF nanocomposites.

Sample	T_g_ (°C)	T_c_ (°C)	T_cc_ (°C)	T_m1_ (°C)	T_m2_ (°C)	∆H_c_ (J/g)	∆H_cc_ (J/g)	∆H_m_ (J/g)	X_c_ (%)
Neat PLA	50.4	106.1	91.3	143.5	152.1	1.3	33.4	35.6	2.3
PLA/CNF1	51.4	111.2	80.1	141.4	151.1	19.5	11.1	37.7	28.4
PLA/CNF2	47.1	114.1	74.9	136.4	147.0	22.2	11.5	40.8	31.2
PLA/CNF3	43.9	114.6	71.0	136.7	147.1	33.6	0.9	42.3	44.2
PLA/CNF4	40.9	110.1	75.2	131.4	143.5	29.7	2.6	43.0	43.1
PLA/CNF5	37.3	106.4	73.6	126.4	140.8	28.1	3.6	37.2	35.9
PLA/CNF6	36.8	102.7	70.3	121.4	135.4	21.4	6.4	30.9	26.1

**Table 2:** Avrami parameters for isothermal crystallization of PLA/CNF nanocomposites.

Sample	T_c_ = 90 °C				T_c_ = 100 °C				T_c_ = 110 °C			
	n	k (min^−n^)	t_1/2_ (min)	1/ t_1/2_ (min^−1^)	n	k (min^−n^)	t_1/2_ (min)	1/ t_1/2_ (min^−1^)	n	k (min^−n^)	t_1/2_ (min)	1/ t_1/2_ (min^−1^)
Neat PLA	3.22	5.72 × 10^−8^	158.01	0.006	2.74	2.83 × 10^−6^	92.72	0.011	2.46	2.46 × 10^−6^	165.59	0.006
PLA/CNF1	3.25	1.35× 10^−4^	13.85	0.072	3.13	2.60 × 10^−4^	12.38	0.081	3.14	5.26 × 10^−6^	42.72	0.023
PLA/CNF2	3.10	6.80 × 10^−4^	9.35	0.107	3.26	6.51 × 10^−4^	8.49	0.118	2.78	3.35 × 10^−5^	35.87	0.028
PLA/CNF3	2.59	1.09 × 10^−2^	4.99	0.200	3.11	2.45 × 10^−1^	1.40	0.716	3.34	1.20 × 10^−5^	26.63	0.038
PLA/CNF4	3.53	4.69 × 10^−4^	7.92	0.126	3.20	1.39 × 10^−3^	6.97	0.144	2.89	3.09 × 10^−5^	31.85	0.031
PLA/CNF5	3.44	2.88 × 10^−4^	9.64	0.104	3.36	2.88 × 10^−4^	10.16	0.098	3.15	1.09 × 10^−5^	33.54	0.030
PLA/CNF6	2.94	3.31 × 10^−4^	13.45	0.074	3.31	1.55 × 10^−4^	12.59	0.079	2.64	5.97 × 10^−5^	34.60	0.029

**Table 3:** Mechanical properties of neat PLA and PLA/CNF nanocomposites.

Composition	Tensile Strength (MPa)	Young’s Modulus (GPa)
Neat PLA	70.6 ± 0.3 ^f^	2.9 ± 0.0 ^e^
PLA/CNF1	72.5 ± 0.4 ^d^	3.1 ± 0.1 ^c,d^
PLA/CNF2	73.6 ± 0.4 ^c^	3.2 ± 0.0 ^b^
PLA/CNF3	74.1 ± 0.5 ^b^	3.3 ± 0.1 ^a^
PLA/CNF4	76.1 ± 0.1 ^a^	3.3 ± 0.0 ^a^
PLA/CNF5	71.3 ± 0.5 ^e^	3.1 ± 0.0 ^c^
PLA/CNF6	68.5 ± 0.5 ^g^	3.0 ± 0.0 ^d^

All data are means of 5 replicates ± S.D. The superscript letters indicate significant difference (*p* < 0.05) according to Duncan’s multiple range test.

**Table 4:** PLA crystallite size in nanocomposites at various CNF loading.

Sample	2θ (degree)	β (degree)	D (nm)
Neat PLA	17.10	10.55	0.761
PLA/CNF1	16.93	10.71	0.750
PLA/CNF2	16.87	10.78	0.745
PLA/CNF3	17.35	10.12	0.794
PLA/CNF4	17.03	10.61	0.757
PLA/CNF5	17.50	9.94	0.809
PLA/CNF6	17.22	10.48	0.766
